# Supplementary material for: Predictors of Mental Health Service Utilization among Frontline Healthcare Workers during the COVID-19 Pandemic
Source: Int J Environ Res Public Health. 2023 Mar 30;20(7):5326. doi: 10.3390/ijerph20075326 (PMC10094311; doi:10.3390/ijerph20075326)
Supplement: Supplementary file 1 [file ijerph-20-05326-s001.zip › ijerph-2208874-supplementary.pdf]

Supplemental Table S1. Study measures grouped based on Andersen's Behavioral Model of Health Services Use.

**Predisposing Factors**	
Sociodemographic and occupational characteristics	Standard questions were used to assess basic sociodemographics (e.g., age, gender, race/ethnicity) and occupational characteristics (e.g., profession, years in practice, supervisory role).
History of mental disorder	History of mental disorder was assessed with the following question: Have you ever been diagnosed by a doctor or healthcare professional with clinical depression, an anxiety disorder, posttraumatic stress disorder, or another mental health condition?
Perceived preparedness	Sum of affirmative responses to the following questions (assessed using No vs. Yes response options):<br>1. My work and activities before the coronavirus pandemic provided me with helpful training to perform my current clinical work<br>2. In my current clinical setting, I am adequately informed about my clinical duties and the role I am expected to play<br>3. At present, I have a good idea of how long my current level/volume of work will last.<br>4. I am adequately trained to perform the professional tasks required of me during this pandemic.
Acute and post-acute surge stressors	Composite measures of pandemic-related stressors at T1 and T2[1] were assessed using questions assessing number of hours worked onsite, number of COVID-19 patients assessed/treated, redeployment status during pandemic, perceived personal medical risk for COVID-19-related complications, number of coworkers infected with COVID-19, having a coworker hospitalized or in ICU to treat COVID-19, having a coworker who died from COVID-19-related complications, not having enough personal protective equipment, not having enough testing for staff and patients, occupational exposures to COVID-19 patients, and personal exposures to COVID-19 infection.
Infection-, family-, and work-related concerns	Composite measures were computed to summarize items assessing infection- (e.g., Worry about infecting patients with COVID-19), family- (e.g., Feel torn between desire/duty to help patients versus loved ones (family, friends, etc.)), and work-related (Worry about not being able to do enough for COVID-19 patients) concerns during the pandemic.[2]
Relationship and work difficulties	The Brief Inventory of Psychosocial Functioning (B-IPF[7]) was used to assess work difficulties (e.g., "I had trouble at work") and relationship difficulties (e.g., "I had trouble with my family relationships").
**Enabling Factors**	
Feel valued/supported at work	Sum of standardized scores on the following questions (Assessed on 4-point scale: Not at all valued, Slightly valued, Moderately valued, Very much valued):

	In your opinion, to what extent do you feel valued by:<br>1. Your immediate supervisors (team leader, service chief, etc.)<br>2. Hospital leadership<br><br>In your opinion, what is the current level of: (Assessed on 3-point scale: Low, Medium, High):<br>1. Camaraderie/team spirit among your group of co-workers in your own clinical practice team or setting.<br>Support from your hospital leadership.
Perceived social support	Score on abbreviated 3-item version of the Medical Outcomes Study Social Support Scale[15] (Assessed on 5-point scale: None of the time, A little of the time, Some of the time, Most of the time, All of the time).<br><br>How often is each of the following kinds of support available to you if you need it?<br>1. Someone to love you and make you feel wanted (i.e., emotional support)<br>2. Someone to help you if you were confined to bed (i.e., instrumental support)<br>Someone to give you good advice in a crisis (i.e., appraisal support)
**Need-based Factors**	2.
MDD symptoms	3. Score on the Patient Health Questionnaire-8[3], an eight-item measure that assesses symptoms of MDD experienced over the previous 2 weeks. A positive screen for significant MDD symptoms was defined by a score ≥ 10, which yields comparable estimates of the prevalence of current major depressive or other depressive disorders relative to diagnostic interviews.
GAD symptoms	Score on the Generalized Anxiety Disorder-7[4], a seven-item measure that assesses symptoms of GAD experienced over the past 2 weeks. A positive screen for significant GAD symptoms was defined by a score ≥10, which has a sensitivity of 0.89 and specificity of 0.82 in diagnosing GAD (24).
PTSD symptoms	Scores on a 4-item PTSD-Checklist[5], an abbreviated version of the PTSD-Checklist for DSM-5 (PCL-5; 22), with questions modified to assess PTSD symptoms related to COVID-19 exposure (e.g., “Over the past two weeks, how often were you bothered by repeated, disturbing, and unwanted memories of your experiences related to the COVID-19 pandemic?") A positive screen for significant COVID-19-related PTSD symptoms was defined by a score ≥ 8, which showed the highest efficiency (90.4%; sensitivity=0.81, specificity=0.94) in diagnosing PTSD.
Burnout	Burnout was assessed using a slightly modified single-item Mini-Z measure[6], which is rated on a 5-point scale and asks participants to choose the best answer (1-5) as it relates to their work

	experience.[14] Options are "I enjoy my work. I have no symptoms of burnout" (score of 1) to "I feel completely burnt out. I am at the point where I may need to seek help." (score of 5). This item was modified to include a definition burnout, as follows: "Burnout is a long-term stress reaction characterized by depersonalization, including cynical or negative attitudes toward patients, emotional exhaustion, a feeling of decreased personal achievement and a lack of empathy for patients." A positive screen for burnout was operationalized as a score of 3 ("I am definitely burning out and have one or more symptoms of burnout, e.g., emotional exhaustion"); 4 ("The symptoms of burnout that I am experiencing won't go away. I think about work frustrations a lot"); or 5 ("I feel completely burned out. I am at the point where I may need to seek help").
Positive emotions	Score on the positive affect subscale of the Positive and Negative Affect Schedule-Short Form (PANAS-SF[8]), which assesses 10 positive emotions: interested, excited, strong, enthusiastic, proud, alert, inspired, determined, attentive, active.
Perceived resilience	Score on the Connor-Davidson Resilience Scale-2 (CD-RISC2)[9]; Responses on 5-point scale ranging from Not true at all to True nearly all the time): I am able to adapt when changes occur; I tend to bounce back after illness, injury
Protective psychosocial characteristics	Factor score of the following measures:<br><br>Items assessing dispositional gratitude, optimism, curiosity/exploration, purpose in life, and religiosity/spirituality (Responses on 7-point scale ranging from Strongly Disagree to Strongly Agree):<br><br>2. I have so much in life to be thankful for.[10]<br>3. In uncertain times, I usually expect the best.[11]<br>4. I frequently find myself looking for new opportunities to grow as a person (e.g., information, people, resources).[12]<br>5. I have discovered clear-cut goals and purpose in my life.[13]<br>In my life, I experience the presence of the Divine (i.e., God).[14]
Restorative behaviors	Sleep hours: "At present, on average how many hours per day do you sleep (out of 24 hours) on a typical workday?<br>Physical exercise: "At this time, during the pandemic, how many days per week do you engage in physical activity (i.e., exercise, sports, yoga, etc.)?"
Self-sufficient coping strategies	Count of engaging in the following coping strategies to help cope with COVID-19-related experiences (adapted from the Brief COPE[16]): planning (e.g., coming up with a strategy for what to do), active

	coping (e.g., taking action to make the situation better), positive reframing (e.g., looking for something positive in what happened), acceptance (e.g., accepting the reality that it happened), humor (e.g., trying to find humor in the situation), religion (e.g., praying, meditating, or finding comfort in spiritual beliefs).
Socially-oriented coping strategies	Count of engaging in the following coping strategies to help cope with COVID-19-related experiences (adapted from the Brief COPE[16]: use of emotional support (e.g., getting comfort or understanding from others), use of instrumental support (e.g., getting advice from others), venting (e.g., expressing negative feelings).
Avoidance coping strategies	Count of engaging in the following coping strategies to help cope with COVID-19-related experiences (from the Brief COPE[16]): self-distraction (e.g., turning to work or other activities to get mind off things), denial (e.g., refusing to believe that it happened), substance use (e.g., using alcohol, nicotine, or drugs to help get through it), behavioral disengagement (e.g., giving up in trying to deal with it), self-blame (e.g., blaming or criticizing myself for what happened).
Distress related to systemic racism	Response to the question: "To what extent have you felt emotionally affected or distressed by systemic racism highlighted by recent events across the country?" rated on a 5-point scale, ranging from Not at all to Extremely.
Distress related to racial disparities in COVID-19 health outcomes	Response to the question: "To what extent have you felt emotionally affected or distressed by the racial disparities in COVID-19-related health outcomes? rated on a 5-point scale, ranging from Not at all to Extremely.

Supplemental Table S2. Characteristics of the sample by mental health treatment status in COVID-19 frontline health care workers.

	**Full Sample N=780**	**No Tx (1) n =631 (80.9%)**	**Stopped Tx (2) n=27 (3.5%)**	**New Tx (3) n=33 (4.2%)**	**Cont. Tx (4) N=89 (11.4%)**		
	n(%) or mean (SD)	n(%) or mean (SD)	n(%) or mean (SD)	n(%) or mean (SD)	n(%) or mean (SD)	Test of difference	Pairwise contrasts
**Demographic and Occupational Characteristics**							
**Age**						0.92	--
< 35	462 (59.2%)	370(58.6%)	16 (59.3%)	22 (66.7%)	54 (60.7%)		
≥ 35	318 (40.8%)	261(41.4%)	11 (40.7%)	11 (33.3%)	25 (39.3%)		
**Gender**						5.50	--
Female	567 (72.7%)	450(71.3%)	18 (66.7%)	27 (81.8%)	72 (80.9%)		
Male	213 (27.3%)	181(28.7%)	9 (33.3%)	6 (18.2%)	17 (19.1%)		

**Race/ethnicity**						42.21***	
White, non-Hispanic	414 (53.1%)	309(49.0%)	17 (63.0%)	18 (54.5%)	70 (78.7%)		4>1,3
Asian	197 (25.3%)	176(27.9%)	3 (11.1%)	10 (30.3%)	8 (9.0%)		1,3>4
Black, non-Hispanic	39 (5.0%)	34 (5.4%)	1 (3.7%)	1 (3.0%)	3 (3.4%)		--
Hispanic	46 (5.9%)	41 (6.5%)	0 (0%)	0 (0%)	5 (5.6%)		--
Other, mixed race	30 (3.8%)	23 (3.6%)	3 (11.1%)	2 (6.1%)	2 (2.2%)		2>4
Prefer not to say	54 (6.9%)	48 (7.6%)	3 (11.1%)	2 (6.1%)	1 (1.1%)		2>4
**Relationship Status**						2.27	
Single/divorced/ widowed	214 (27.4%)	167(26.5%)	10 (37.0%)	11 (33.3%)	26 (29.2%)		
Married /partnered	566 (72.6%)	464(73.5%)	17 (63.0%)	22 (66.7%)	63 (70.8%)		
Living with children	232 (29.7%)	202(32.0%)	7 (25.9%)	3 (9.1%)	20 (22.5%)	10.73*	1>3
**Profession**						47.04***	
Registered nurse	265 (34.0%)	225(35.7%)	9 (33.3%)	8 (24.2%)	23 (25.8%)		2>3
Residents/Fellows	184 (23.6%)	144(22.8%)	3 (11.1%)	13 (39.4%)	24 (27.0%)		3>2
Attending MD/DO	181 (23.2%)	143(22.7%)	10 (37.0%)	4 (12.1%)	24 (27.0%)		2>3
PA/NP	112 (14.4%)	99 (15.7%)	4 (14.8%)	5 (15.2%)	4 (4.5%)		1,3>4
Other	38 (4.9%)	20 (3.2%)	1 (3.7%)	3 (9.1%)	14 (15.7%)		4>1
Years in practice	8.3 (8.6)	8.7 (9.0)	6.3 (6.5)	6.3 (6.2)	6.6 (6.6)	2.59	--
History of mental disorder	160 (20.5%)	59 (9.4%)	15 (55.6%)	13 (39.4%)	73 (82.0%)	282.29***	4>2,3>1
Past-year burnout	300 (38.5%)	239(37.9%)	10 (37.0%)	14 (42.4%)	37 (41.6%)	0.68	--
Supervisory role	191 (24.5%)	158(25.1%)	8 (29.6%)	3 (9.1%)	22 (24.7%)	4.73	--
**COVID-19 pandemic-related variables**							
Acute surge stressors	0 (1.0)	0 (1.0)	-0.1 (1.2)	0 (1.2)	0.0 (1.0)	0.04	--
Post-acute surge stressors	0 (1.0)	0 (1.0)	-0.2 (0.7)	0 (1.0)	-0.1 (0.9)	0.47	--
Infection-related concerns	0 (1.0)	-0.1 (0.9)	-0.1 (1.4)	0.1 (0.9)	-0.1 (1.1)	0.46	--
Family-related concerns	0 (1.0)	-0.1 (1.0)	0.1 (1.0)	-0.4 (1.2)	-0.2 (1.0)	1.57	--
Work-related concerns	0 (1.0)	0 (0.9)	-0.2 (1.0)	0.3 (1.0)	0 (0.9)	1.70	--
**Psychopathology, functioning, and racism-related distress**							
Positive screen for MDD symptoms	184 (23.6%)	135(21.4%)	10 (37.0%)	14 (42.4%)	25 (28.1%)	11.89**	3>1

Positive screen for GAD symptoms	179 (22.9%)	135(21.4%)	11 (40.7%)	11 (33.3%)	22 (24.7%)	7.87*	2>1
Positive screen for PTSD symptoms	140 (18.0%)	110(17.4%)	9 (34.6%)	8 (24.2%)	13 (14.6%)	6.57	--
Positive screen for burnout	304 (39.0%)	232(36.8%)	10 (37.0%)	19 (57.6%)	43 (48.3%)	9.40*	3,4>1
Relationship difficulties	21.0 (22.6)	19.6 (22.1)	21.0 (19.3)	32.0 (26.7)	27.3 (23.1)	5.79***	3,4>1
Work difficulties	20.0 (21.9)	18.8 (21.4)	21.5 (18.1)	29.4 (25.9)	24.2 (23.9)	3.73*	3,4>1
Distress related to systemic racism	1.8 (1.2)	1.7 (1.2)	2.2 (1.2)	2.4 (1.2)	2.2 (1.2)	8.57***	3,4>1
Distress related to racial disparities in COVID-19 outcomes	2.1 (1.2)	2.0 (1.2)	2.3 (1.2)	3.0 (0.9)	2.5 (1.1)	10.95***	3,4>1
**Protective psychosocial variables**							
Positive emotions	32.0 (7.8)	32.5 (7.6)	30.8 (8.1)	26.7 (7.8)	30.3 (8.1)	7.96***	1>3,4
Perceived resilience	6.5 (1.3)	6.6 (1.3)	6.7 (1.1)	5.9 (1.3)	6.1 (1.3)	7.38***	1>3,4
Protective psychosocial characteristics	0 (1.0)	0 (1.0)	0.2 (0.8)	-0.7 (1.3)	-0.4 (1.0)	11.61***	1>3,4; 2>3
Feel valued/supported at work	0 (1.0)	0.1 (0.8)	0.2 (0.5)	0.2 (0.6)	0.1 (0.8)	0.59	--
Perceived social support	12.3 (2.9)	12.3 (2.8)	12.3 (2.5)	11.2 (3.54)	12.6 (2.62)	2.18	--
**Restorative behaviors**							
Sleep hours	6.4 (1.1)	6.4 (1.1)	6.8 (1.2)	6.6 (1.1)	6.8 (1.1)	5.57***	4>1
Physical exercise	2.2 (1.9)	2.2 (1.9)	2.5 (1.8)	2.3 (2.0)	2.4 (2.0)	0.58	--
**Coping strategies**							
Self-sufficient coping	1.4 (1.0)	1.5 (1.1)	1.4 (1.0)	1.1 (1.2)	1.1 (1.0)	3.80*	1>4
Socially supported coping	1.0 (0.7)	1.0 (0.7)	1.0 (0.8)	1.0 (0.5)	1.1 (0.7)	1.95	--
Avoidant coping	0.8 (0.6)	0.8 (0.6)	0.7 (0.5)	1.0 (0.8)	0.9 (0.7)	3.70*	3,4>1

References

1. Feingold, J.H.; Peccoralo, L.; Chan, C.C.; Kaplan, C.A.; Kaye-Kauderer, H.; Charney, D.; Verity, J.; Hurtado, A.; Burka, L.; Syed, S.A.; et al. Psychological Impact of the COVID-19 Pandemic on Frontline Health Care Workers During the Pandemic Surge in New York City. *Chronic Stress* **2021**, *5*, https://doi.org/10.1177/2470547020977891.
2. Norman, S.B.; Feingold, J.H.; Kaye-Kauderer, H.; Kaplan, C.A.; Hurtado, A.; Kachadourian, L.; Feder, A.; Murrough, J.W.; Charney, D.; Southwick, S.M.; et al. Moral distress in frontline healthcare workers in the initial epicenter of the COVID-19 pandemic in the United States: Relationship to PTSD symptoms, burnout, and psychosocial functioning. *Depression Anxiety* **2021**, *38*, 1007–1017, https://doi.org/10.1002/da.23205.

3. Kroenke, K.; Strine, T.W.; Spitzer, R.L.; Williams, J.B.; Berry, J.T.; Mokdad, A.H. The PHQ-8 as a measure of current depression in the general population. *J. Affect. Disord.* **2009**, *114*, 163–173, https://doi.org/10.1016/j.jad.2008.06.026.
4. Spitzer, R.L.; Kroenke, K.; Williams, J.B.W.; Löwe, B. A Brief Measure for Assessing Generalized Anxiety Disorder: The GAD-7: The GAD-7. *Arch. Intern. Med.* **2006**, *166*, 1092–1097, doi:10.1001/archinte.166.10.1092.
5. Geier, T.J.; Hunt, J.C.; Hanson, J.L.; Heyrman, K.; Larsen, S.E.; Brasel, K.J.; Deroon-Cassini, T.A. Validation of Abbreviated Four- and Eight-Item Versions of the PTSD Checklist for*DSM-5*in a Traumatically Injured Sample. *J. Trauma. Stress* **2020**, *33*, 218–226, https://doi.org/10.1002/jts.22478.
6. Rohland, B.M.; Kruse, G.R.; Rohrer, J.E. Validation of a single-item measure of burnout against the Maslach Burnout Inventory among physicians. *Stress Heal.* **2004**, *20*, 75–79, https://doi.org/10.1002/smi.1002.
7. Kleiman, S.E.; Bovin, M.J.; Black, S.K.; Rodriguez, P.; Brown, L.G.; Brown, M.E.; Lunney, C.A.; Weathers, F.W.; Schnurr, P.P.; Spira, J.; et al. Psychometric properties of a brief measure of posttraumatic stress disorder–related impairment: The Brief Inventory of Psychosocial Functioning.. *Psychol. Serv.* **2020**, *17*, 187–194, https://doi.org/10.1037/ser0000306.
8. Watson, D.; Clark L.A.; Tellegen, A. Development and Validation of Brief Measures of Positive and Negative Affect: The PANAS Scales. *J Pers Soc Psychol.* **1988**, *6*, 1063–1070.
9. Vaishnavi, S.; Connor, K.; Davidson, J.R. An abbreviated version of the Connor-Davidson Resilience Scale (CD-RISC), the CD-RISC2: Psychometric properties and applications in psychopharmacological trials. *Psychiatry Res.* **2007**, *152*, 293–297, https://doi.org/10.1016/j.psychres.2007.01.006.
10. McCullough, M.E.; Emmons, R.A.; Tsang, J.A. The Grateful Disposition: A Conceptual and Empirical Topography. *J Pers Soc Psychol.* **2002**, *82*,112–127.
11. Scheier, M.F.; Carver, C.S.; Bridges, M.W. Distinguishing Optimism from Neuroticism (and Trait Anxiety, Self-Mastery, and Self-esteem): A Re-evaluation of the Life Orientation Test. *J Pers Soc Psychol.* **1994**, *67*,1063–1078.
12. Kashdan, T.B.; Gallagher, M.W.; Silvia, P.J.; Winterstein, B.P.; Breen, W.E.; Terhar, D.; Steger, M.F. The curiosity and exploration inventory-II: Development, factor structure, and psychometrics. *J. Res. Pers.* **2009**, *43*, 987–998, https://doi.org/10.1016/j.jrp.2009.04.011.
13. Schulenberg, S.E.; Schnetzer, L.W.; Buchanan, E.M. The Purpose in Life Test-Short Form: Development and Psychometric Support. *J. Happiness Stud.* **2010**, *12*, 861–876, https://doi.org/10.1007/s10902-010-9231-9.
14. Koenig, H.G.; Büssing, A. The Duke University Religion Index (DUREL): A Five-Item Measure for Use in Epidemological Studies. *Religions* **2010**, *1*, 78–85. https://doi.org/10.3390/rel1010078
15. Sherbourne, C.D.; Stewart, A.L. The MOS social support survey. *Soc. Sci. Med.* **1991**, *32*, 705–714, https://doi.org/10.1016/0277-9536(91)90150-b.
16. Carver, C.S. You want to measure coping but your protocol's too long: Consider the Brief COPE. *Int. J. Behav. Med.* **1997**, *4*, 92–100., doi:10.1207/s15327558ijbm0401_6.
